# Application of NGS Technology in Understanding the Pathology of Autoimmune Diseases

**DOI:** 10.3390/jcm10153334

**Published:** 2021-07-28

**Authors:** Anna Wajda, Larysa Sivitskaya, Agnieszka Paradowska-Gorycka

**Affiliations:** 1 Department of Molecular Biology, National Institute of Geriatrics, Rheumatology and Rehabilitation, 02-637 Warsaw, Poland; annawajda2046@gmail.com (A.W.); paradowska_aga@interia.pl (A.P.-G.); 2 Institute of Genetics and Cytology, National Academy of Sciences of Belarus, 220072 Minsk, Belarus

**Keywords:** next-generation sequencing, autoimmune diseases, autoimmune connective tissue diseases, HLA, microRNA, microbiome

## Abstract

NGS technologies have transformed clinical diagnostics and broadly used from neonatal emergencies to adult conditions where the diagnosis cannot be made based on clinical symptoms. Autoimmune diseases reveal complicate molecular background and traditional methods could not fully capture them. Certainly, NGS technologies meet the needs of modern exploratory research, diagnostic and pharmacotherapy. Therefore, the main purpose of this review was to briefly present the application of NGS technology used in recent years in the understanding of autoimmune diseases paying particular attention to autoimmune connective tissue diseases. The main issues are presented in four parts: (a) panels, whole-genome and -exome sequencing (WGS and WES) in diagnostic, (b) Human leukocyte antigens (HLA) as a diagnostic tool, (c) RNAseq, (d) microRNA and (f) microbiome. Although all these areas of research are extensive, it seems that epigenetic impact on the development of systemic autoimmune diseases will set trends for future studies on this area.

## 1. Introduction

High-throughput nucleic acid sequencing methods are a breakthrough in research based on molecular biology. Possibility of generation of a massive pool of data has changed the scientific approach in basic, applied and clinical research. ‘First-generation’ technology is referred to as the automated Sanger method whereas newer methods which may use different sequencing technologies and variable platforms are called next-generation sequencing (NGS) or deep sequencing [1]. ‘Third-generation’ of sequencing includes the use of scanning tunneling electron microscope (TEM), fluorescence resonance energy transfer (FRET), single-molecule detection, and protein nanopores [2]. ‘Fourth-generation’ technology eliminates optical detection and the nucleotide bases are labeled with distinct heavy atoms to distinguish bases [3] or measures the density of electron flow through a scanning tip [4].

Although the concept of Sanger and NGS sequencing technology is similar, with fluorescently tagged nucleotides added onto an amplified DNA template by DNA polymerase, the main difference between those two methods is the sequencing volume. In Sanger method, only a single DNA fragment may be sequenced at a time, NGS allow sequencing of millions of fragments per run. Therefore, deep sequencing more likely allows detecting of novel and rare variants. Nevertheless, Sanger sequencing can be still recommended for the analysis of small regions and study on limited number of samples or genomic targets.

Frederick Sanger started his research on sequencing of nucleic acid around 1960, in 1977 appeared an article which described newly developed method based on using small amounts of dideoxynucleotides [5]. Over the years, technology of sequencing has been developed and gained importance in many fields of life science. Certainly a race in the Human Genome Project triggered development of NGS [6,7]. First NGS technology was based on pyrosequencing method by 454 Life Sciences (now Roche) in 2005. In this technology, incorporation of nucleotide is associated with releasing of pyrophosphate which leads to monitored in real time light emission [8]. In 2008, first time used high-throughput DNA sequencing results has been published in Nature. This analysis of James Watson’s DNA, as a part of Human Genome Project, revealed novel genes not previously identified by traditional genomic sequencing. Furthermore, this technology confirmed the results of sequencing by traditional method but appeared to be faster and cheaper. Additionally it avoided the arbitrary loss of genomic sequences inherent in random shotgun sequencing by bacterial cloning because it amplifies DNA in a cell-free system [9]. Nowadays, novel NGS applications allow analyzing not only sequencing of DNA in traditional meaning but also analysis of the interaction between protein-DNA (CHIP-seq) [10], analysis of gene expression without knowledge of the targeted genes (RNA-seq) [11], RNA–protein interactions CLIP-seq [12], RNA–DNA interactions (CHART) [13], RNA-chromatin interaction [14], and DNA methylation [15]. Different platforms are available to date including Roche, Ion Torrent (Thermofisher), Illumina, Solid, PacBio, Helicose, Oxford Nanopore, Qiagen and BGI etc. Ion Torrent Personal Genome Machine (PGM) does not rely on the optical detection of incorporated nucleotides using fluorescence and camera scanning but employs semiconductor technology to measure pH changes in polymerization events [16].

NGS approaches are used in the study on drug resistance [17], cancer research [18] and many others. The aim of this review is to present the recent scope of NGS analysis in the aspect of autoimmune connective tissue diseases. These entities, a part of autoimmune diseases family represented by at least 80 different maladies [19], are characterized by continuous inflammatory process in the joints, ligaments, bones, and muscles as a consequence leading to their irreversible degradation [20]. Systemic forms of inflammation cause damage to virtually any bodily organ or system, including the heart, kidneys, lungs, blood vessels, eyes, and skin. Prolonged destruction of these organs leads to significant mortality. Therefore in clinical practice autoimmune diseases require a holistic, interdisciplinary approach. Injuries/Destruction process is the result of T cell activities [21], disturbed T cell subtypes homeostasis [22,23,24,25]. The immune activation may persist after the infection and then whereas any microbial antigen cannot be detected [26]. Furthermore, immune complexes with DNA, RNA, citrullinated proteins, B and T cell epitopes underlie Toll-like receptor (TLR) activation and trigger the inflammatory pathways [27,28]. The molecular mechanisms of the autoimmune diseases pathogenesis are not yet completely clear [26]. Furthermore, in case of connective tissue diseases (CTD), once diagnosed autoimmune CTD may evolve into another. It has happened in case of mixed connective tissue disease (MCTD) and systemic lupus erythematosus (SLE) or systemic sclerosis (SSc) [29]. Few years of observation of the patients with undifferentiated connective tissue disease (UCTD) demonstrated the most frequent development into RA, SLE, Sjogren’s syndrome, scleroderma or MCTD [30,31] Profesor Shoenfeld with his colleagues use the term “kaleidoscope of autoimmunity” which describes the possible shift of one disease to another or co-existence of few autoimmune diseases in a patient or in the same family [32]. Humbert and Dupond in 1988 described for the first time Multiple autoimmune syndromes (MAS) as a coexistence of three or more different ADs in a single patient. MAS has been divided into three types, where Type II includes Sjögren’s syndrome (SS), RA, primary biliary cirrhosis, scleroderma, and autoimmune thyroid disorders [33]. Currently, polyautoimmunity term is commonly used. This phenomenon is explained by shared similar immunogenetic and pathologic mechanisms [34]. On the one hand, complex genetic and epigenetic factors involved in the pathogenesis and clinical course of the disease may obscure the diagnostic process, affect the clinical picture and its qualification. On the other hand, it seems that high-throughput research methods, including NGS, are necessary in the case of basic research and may give more possibilities for diagnostics and individualized therapy. Areas of exploration range are really broad, from sequencing of selected genes, to comprehensive analysis of microRNA expression or transcriptome, to sequencing and describing variety in the microbiome with correlation to the disease entities. 

In Table 1 some selected studies on autoimmune connective tissue conducted within the last 5 years using NGS technology are presented. In the present article, we refer to the technological approaches/methods. Clinical NGS applications described in the review are presented in Table 2. For information on NGS methods in the aspect of particular autoimmune diseases, we refer the readers to the H.Niu review [35].

## 2. Panels, Whole-Genome and -Exome Sequencing in Diagnostic

Molecular diagnosis is of major importance for treatment, prognosis, and genetic counseling. NGS technologies have transformed clinical diagnostics and are broadly used from neonatal emergencies to adult conditions where the diagnosis cannot be made based on clinical symptoms. 

Recently, Boegel et al. analyzed usage of highthroughput sequencing in rheumatic research. According to the authors, the most studied entities among the rheumatic diseases are rheumatoid arthritis, systemic lupus erythematous and osteoarthritis (OA). Whole exome sequencing (WES) and target panels are estimated to consist about 24% of applications used in high throughput methods used in research in rheumatology [72]. However, especially WES reveals high diagnostic potential. 

Targeted gene panels still have some benefits in clinical application, such as reduced cost, easier interpretative challenges and shorter time for results given. The usefulness of a targeted NGS approach in monogenic auto-inflammatory disorders (AID) and vasculitis diagnosis was demonstrated by Omoyinmi et al. A panel of 166 genes accurately detected disease-causing variants in 22 AID patients with previously validated mutations in a variety of the genes; and in 16 patients with suspected AID in whom previous Sanger-based genetic screening had been non-diagnostic. A diagnosis was reached in 32% of participants [54]. Rusmini et al. developed and evaluated an NGS-based protocol for simultaneous screening of 10 genes to improve the diagnosis and genotype interpretation of systemic AIDs. As a result, the overall clinical picture of 34 patients tested with the gene panel was re-evaluated and pathogenic variants were not found in several patients with a typical AID phenotype. Moreover, some patients with previously genetically confirmed AID were carriers of additional mutations in other genes that could contribute to the clinical presentation. No pathogenic variants have been found in several cases of undefined systemic AID allowing some diagnoses to be ruled out [50].

In recent years, there has been an increasing amount of case reports reported, which may be useful for clinicians. For example, Mauro et al. diagnosed PTEN hamartoma tumor syndromes (PHTS) caused by sporadic heterozygous mutation in phosphatase and tensin homolog (PTEN) in a 3-year-old boy by using an NGS panel. The authors designed a diagnostic panel using The Agilent EArray online tool. Overall, 166 genes broadly associated with immune dysregulation or vasculopathy, the vasculitis and inflammation were targeted [51]. For the first time, the association between tartrate-resistant acid phosphatase activity and interferon metabolism has been identified thanks to the targeted sequencing of the tartrate-resistant acid phosphatase gene (ACP5). Biallelic mutations in ACP5 have been described in SLE, Sjögren’s syndrome, hemolytic anemia, thrombocytopenia, hypothyroidism, inflammatory myositis, Raynaud’s disease and vitiligo. Moreover, regardless of the disease entity, all subjects were characterized by elevated serum interferon-alpha (IFN-α) activity, and gene expression profiling in whole blood defined a type I interferon signature [52]. Based on a targeted panel designed to selectively capture known genes associated with different forms of calcium/parathormone metabolic disorders, novel pathogenic variants of the autoimmune regulator (AIRE) gene in Autoimmune Polyendocrine Syndrome Type I with Atypical Presentation have been identified by Cinque et al. [53].

Another advantage of gene panels is the increased depth of coverage, which allows the detection of mosaicism in targeted sequences. NGS detection of mosaicism depends on the number of reads at the position and the thresholds for calling a variant. Omoyinmi et al. used a sensitive bioinformatics pipeline to determine low-level mosaicism (3%) for NLR family pyrin domain containing 3 (NLRP3) mutation in Cryopyrin-associated periodic syndrome (CAPS) patient and recommended manually checking the sequence alignment file (BAM) of the gene if mosaicism is suspected [54]. Extremely low (nucleotide-binding oligomerization domain containing 2 (NOD2) mosaicism was verified in a patient with the mild phenotype of Blau syndrome. The amplicon-based deep sequencing determined 0.9–12.9% mosaicism in different tissues, where the depth of coverage of the NOD2 targeted sequences was 5503–7418 reads [55]. 

Although targeted panels have obvious benefits, the WES has become the most popular NGS approach in molecular diagnostics. The main disadvantage of gene panels is not all disease-related genes have been identified and included in them. At this point of view, WES provides coverage of all coding parts of the human genome and reveals high diagnostic potential. Approximately 85% of disease-related mutations are found in the exome [73]. This method can be used to identify rare or novel deleterious variants as genetic causes of disease and may be to the treatment implication like in the case described by Ellyard et al. The authors performed WES in a 4 year old girl with early onset SLE and discovered a rare, homozygous mutation in the three prime repair exonuclease 1 gene (TREX1) that was predicted to be highly deleterious. The TREX1 R97H mutant protein was characterized by high reduction in exonuclease activity and was associated with an increased IFN-α signature in the SLE patient. Based on this study the patient is now a candidate for neutralizing anti- IFNα therapy [56]. Mitsunaga (2013) successfully applied WES to uncover rare variants in butyrophilin-like 2 gene (BTNL2) associated with RA [57].

Delgado-Vega et al. sequenced the exome of the most distantly related patients with SLE from two well-studied multi-case SLE families from Iceland [58]. The authors identified 19 non-synonymous single-nucleotide variants (SNV) presented in heterozygous state in the exome-sequenced patients. In this study none of the identified genes affected stop gain, stop loss, nonsense, or essential splice variant. Nevertheless, the authors restricted pool of analyzed genes by favoring the best-quality SNVs shared by all the affected sequenced individuals from each family. Additionally, SNVs revealed a very low allele frequency (minor allele frequency (MAF) < 0.01) in an internal control population. Moreover, analyzed genes were must have easily recognized functional consequences (gain or loss of a stop codon, nonsense, missense, and essential splice sites). A total of nineteen variants passed all quality-, annotation- and frequency-based filtering criteria, and were validated by SEQUENOM and/or Sanger sequencing. Indicated changes in some genes, e.g., Charcot-Leyden crystal protein (CLC, rs146776010), F-box and leucine-rich repeat protein 14 (FBXL14, rs117331652), DNA cross-link repair protein 1C (DCLRE1C, rs772438042) or NOTCH receptor 1 (NOTCH1, rs758642073), may have direct impact on immunology system and possible development of autoimmune disorder. For example CLC is associated with regulation of T cell anergy, regulation of T cell cytokine production, regulation of activated T cell proliferation. Among FBXL14 gene related pathways are innate immune system and Class I MHC mediated antigen processing and presentation. Diseases associated with DCLRE1C include severe combined immunodeficiency and Omenn Syndrome [58]. 

Nevertheless, there are technical limitations of WES in molecular diagnostics. First, this approach focuses on the coding regions and nearby intronic sequences where canonical splice sites are located [73]. As a result, pathogenic variants cannot be detected in regulatory regions or deep intron sequences [74]. Second, it is not easy to detect copy number variations (CNVs) and to establish accurate breakpoints for deletions and duplications [75,76]. Besides, there are difficulties in the detection of the low-level mosaicism, imprinting and uniparental disomy [73]. Reliable detection of these genomic pathogenic contributions requires special bioinformatics pipelines, manual inspection of NGS data or alternative molecular approaches [76]. Third, the most recognized disadvantage of WES is a higher rate of ambiguous results, including variants of unknown significance (VUS) and variants in genes with limited medical management [77]. Both of these findings lead to a lack of clear recommendations for the diagnosis and treatment of patients. 

Some of these limitations can be solved with whole genome sequencing (WGS). WGS covers coding and non-coding regions and captures the full spectrum of genomic pathogenic contribution to human disease. But there is another challenge in the clinical application of WGS. This type of testing identifies a large number of genetic variants. As a result, the manual stage of interpretation and searching for disease-causing variants becomes laborious and time-consuming [77]. 

The most recognized advantage of WGS is ability to detect complex repeat expansions, CNVs and structural variants (SVs). CNVs associated with human pathologies range from chromosomal aneuploidy, to microduplication and microdeletion syndromes, and include smaller SVs that affect single genes and exons [78]. Some CNVs loci are commonly implicated in various autoimmune diseases, such as Fcγ receptors in patients with systemic lupus erythematosus or idiopathic thrombocytopenic purpura and β-defensin genes in patients with psoriasis or Crohn’s disease [79,80,81]. Another study that compared whole-genome sequence in monozygotic twins revealed that CNVs may be associated with SLE [59]. Only the genes with discordant copy number losses in SLE patient were significantly enriched in pathways involved in this disease development. Enriched SLE pathway genes with copy number losses in twin with SLE were histone cluster 2 H2A family member A3 (HIST2H2AA3), HIST2H2AA4, HIST2H3A, HIST2H3C, HIST2H4A and HIST2H4B. However, not all genomic regions were completely sequenced. Sequencing coverage of this study had no sufficient power to identify de novo variants linked with SLE. Therefore, based on WGS only 1 putative discordant exonic variant in GGT1 gene (recorded in the Online Mendelian Inheritance in Man database) between the twins was indicated [59].

Almlöf et al. detected by WGS a significant enrichment of ultra-rare (≤0.1%) missense and nonsense mutations in 22 genes known to cause monogenic forms of SLE and seven ultra-rare, coding heterozygous variants in five genes (C1S, DNASE1L3, DNASE1, IFIH1, and RNASEH2A) involved in monogenic SLE. Homozygous nonsense mutation in the C1QC (Complement C1q C Chain) gene was also among identified genes which explains the immunodeficiency and severe SLE phenotype of that patient [60]. Deficiency of early components of the classical pathway (C1, C2 and C4) leads to autoimmunity whereas deficiency of C3 and its regulators has been associated with severe recurrent bacterial infections and autoimmunity. Particularly, SLE may be a clinical consequence of inherited defects in the complement system. Sequence of the complement system in SLE patients may be compared to the sequences with computer databases. The European Society for Immunodeficiencies (ESID) and European Reference Network on Rare Primary Immunodeficiency, Autoinflammatory and Autoimmune Diseases (ERN RITA) recommends clinical-exome and whole-exome sequencing as potentially useful methods [82]. Alternation in genes coding early complement proteins has been revealed in SLE cases with disease onset ≤5 years of age and family history consistent with an autosomal recessive inheritance. This study was performed by WES approach. Authors demonstrated also changes in DNASE1L3 and HDAC7 (histone decetylase 7) and suggested that monogenic causes/associations should be sought in early-onset and/or familial SLE [61]. Based on WES in children with SLE with lymphoproliferation, Li et al. demonstrated a novel phenotype of somatic mutations in the NRAS gene and germline mutations in the PI3CKD gene. According to the authors, these genes together with TNFAIP3 should be considered as candidates gene for testing in children with SLE with lymphoproliferation, particularly for male patients with renal and hematologic involvement and recurrent fevers [62].

Clinical application of WES and WGS become increasingly popular and available, because they can provide diagnosis in cases where the putative causative gene was not suspected or even not known.

## 3. Human Leukocyte Antigens (HLA) as a Diagnostic Tool

Another diagnostic potential in autoimmune diseases revealed Human leukocyte antigens (HLA) sequencing. HLA genes in the major histocompatibility complex (MHC) are thought to increase susceptibility to autoimmune diseases, such as RA [83]. Classic class I MHC molecules are encoded by the HLA-A, -B and -C genes, while non-classical MHC molecules are encoded by the HLA-E, -F, -G, MICA and MICB genes. Non-classical class I MHC molecules can also be encoded outside the MHC region (e.g., CD1). Classic MHC class II molecules are encoded by genes lying in the HLA-DP, -DQ and -DR regions, while non-classical ones—HLA-DM and HLA-DO [84,85,86]. Moreover, HLA region is the most polymorphic region in the human genome [87]. In certain medical procedures, such as hematopoietic cell transplants and solid organ transplants, NGS, particularly RNA-seq, may become a critical method of HLA typing [88]. 

Study on the Belarusian population with clinically different forms of juvenile idiopatic arthritis confirmed that HLA play a significant role in the genetic predisposition to these entities. It has been estimated that it determines up to 18% of disease risk. Moreover, high-throughput HLA typing revealed that different HLA-patterns are involved into the formation of various Juvenile idiopathic arthritis (JIA) subtypes. DQB1*04:02:01 and DR8-haplotype frequencies were significantly higher in patients with oligoarthritis but not systemic JIA when compared with healthy controls. While DQA1*05:01:01 and DQB1*02:01:01 alleles showed a protective effect against both systemic and oliarticular JIA, DRB1*03:01 allele showed the negative association with systemic JIA [63]. Asparagine (Asp) at HLA-B position 9, and phenyloalanine (Phe) at HLA-DPB1 position 9 predispose to RA development. Valine (Val) and Leucyne (Leu) at HLA-DRB1 position 11 are associated with a risk of a higher rate of radiographic progression and also predispose to RA development [64]. Additionally, using NGS method a significant and independent association of HLA-DRB1 shared epitopes (SE) alleles with the reduced T cell receptor (TCR) repertoire diversity of CD4+ T cells in patients with RA has been shown by Sakurai et al. [42]. Another example is HLA-B27 helpful in the diagnostic of ankylosis spondylitis (AS), sacroileitis associated with psoriasis or with inflammatory bowel disease (IBD) or enthesis-related JIA [89]. Association of HLA-B27 with susceptibility to ankylosis spondylitis has been revealed in 1973 by Brewerton et al. [87]. Interestingly, HLA-B27 is estimated to be found in 5% of the general population, 95% of the AS patients are HLA-B27 positive. Eventually, the above mentioned related diseases are linked to HLA-B27 but at the lower frequency [90]. T cell repertoire next-generation sequencing was used to identify motifs enriched among B27-positive AS patients. Study conducted by Faham et al. provided evidence for the hypothesis that specific antigens are involved in the pathogenesis of AS. Moreover, the majority of motifs were observed in CD8+ T cell and TCRβ chain more frequently TCRα drives the antigen specificity in AS patients. The authors recommend this approach to identify disease-related motifs in other autoimmune diseases [91]. NGS technology allowed to identified of gut bacteria strains associated with the AS (including these strains which are correlated with HLA-B27), and expanded knowledge on the role of bacteria in the pathogenesis of AS [92]. Moreover, 70% of patients with AS develop subclinical gut inflammation and between 5% and 10% of AS patients with inflammatory bowel disease (IBD) [93]. Integration of high-density array-based genotyping, and next-generation sequencing helped to determine that half the risk of T1D comes from position 57 in HLA-DQß1, and most of the other half comes from positions 13 and 71 in HLA-DRß1. The DRß1 alleles and the DQß1 alleles interact with each other to confer a differential risk of type 1 diabetes (T1D) [66]. Profaizer et al. claimed that HLA typing by NGS is superior not only to the existing clinical methods for identifying HLA alleles associated with disease but also may provide safer and more effective pharmacotherapy, like in the case of hypersensitivity of allopurinol, carbamazepine or abacavir [94]. Nevertheless, the implementation of this method in clinical practice involves not only factors such as time and finances, but also the complexity of technology. Essential to the actual DNA sequencing is the library construction process with significant differences in the details of general steps: PCR, amplicon quantification, fragmentation, library preparation and the differences between commercially available kits, size selection and final quantification. Furthermore, clean-up steps are important for the removal of adaptor dimers or other DNA artifacts. Small-size artifacts will also affect quantification results. Additionally, different indexing strategies may also affect the results. Over-loading the flow cell may result in poor template generation and low sequencing quality whereas under-loading results in low data output and inefficient usage of the flow cell [95]. 

## 4. RNA-Seq

Deregulation of gene expression leads to pathological changes and the development of many diseases [96]. Many studies due to their financial and technical restrictions are limited to the analysis of gene expression by qPCR methods. NGS-based RNA-seq is used to sequence the total RNA to detect the change of gene expression but also discover novel miRNAs. Nevertheless, due to the fragile nature of RNA, RNA-seq application is also limited [35]. 

High throughput gene expression analysis, RNA-seq, allowed the comparison of hundred genes which would be impossible with traditional methods. Heruth et al. (2012) described 682 genes expressed only in synovial fibroblasts in RA patients (RASFs) but not in normal synovial fibroblasts (SFs). Moreover, 122 of them were up-regulated and 155 down-regulated genes in RASFs with at least two-fold change compared to the SFs. According to this analysis Cellular Movement, Cell Death, and Tissue Development are the top networks affected by downregulated genes in RASFs compared to normal SFs [97]. Another study (2014) on the synovial fibroblast in RA patients revealed 293 genes differentially expressed in comparison to cells of healthy subjects. The highest significant differences have been noted in the case of genes involved in the anatomical structure development, cell membrane formation and stability, and biological adhesion in the most significant pathways such as Wnt signaling, cell adhesion molecules (CAMs), cytokine-cytokine receptors interactions, calcium signaling, regulation of actin cytoskeleton and focal adhesion [98]. An interesting study was conducted by Mittal et al. (2015) which compared the expression levels of genes in different trimesters of pregnancy in women with RA compared to healthy women. In this study, 256 genes showed a greater than two-fold change in expression during pregnancy compared to baseline levels, with distinct temporal trends through pregnancy. Another 98 genes involved in various biological processes including immune regulation exhibited expression patterns that were differentially associated with pregnancy in the presence or absence of RA. Significant changes in the expression profiles compared to pre-pregnancy were observed during the second trimester and were maintained during the third trimester. Examples of genes involved in immune system processes and defense response that displayed a marked pregnancy-related up-regulation included: olfactomedin 4 (*OLFM4* encodes an extracellular matrix glycoprotein that facilitates cell adhesion), matrix metalloproteinase (*MMP8*), lactotransferrin precursor (*LTF*), human α-defensins (DEFA1, DEFA3, DEFA1B), CRISP3, CAMP, OLR1, LCN2, CD177, ABCA13—lipid transport and CEACAM8. Furthermore, genes involved in mast cell activation and immunoglobulin binding were significantly downregulated in the second and third trimesters. In comparison to healthy women, TBC1D3C, HLA-DRB3, DAAM2,—upregulated during pregnancy in all trimesters whereas EPHB4 seemed to reveal higher fold change in healthy women than pregnant women with RA or RA women before pregnancy [99]. Another example of comprehensive analysis is single-cell transcriptomics. Arazi et al. analyzed kidney, urine and blood samples from patients with lupus nephritis (LN) and healthy individuals. Data of gene expression was obtained form 2736 leukocytes and 145 epithelial cells. This study revealed that urine cells have the potential to serve as surrogates for kidney biopsies in assessing the molecular activation state of subsets of infiltrating leukocytes [100].

RNA-seq might be helpful in the prediction of medical treatment. One of the examples might be whole transcriptome sequencing of RA neutrophils where gene expression profile has been used to characterize disease activity and response to TNF inhibitor (TNFi) therapy. Wright et al. described the correlation between interferon level and response to the treatment. Patients in the IFN-high group achieved a better response to TNFi therapy than patients in the IFN-low group [67]. Whole-blood mRNA analysis conducted by Farutin et al. have shown that a crucial factor of a successful response to TNFi treatment within the first 3 months of therapy may be the differences in innate/adaptive immune cell type composition at baseline and that inability of anti-TNF therapy to suppress adaptive immune-related pathways may contribute to the likelihood of treatment failure in these patients [68]. The integrative approach of transcriptome studies applied to identify disease-specific drug targets are recommended by the authors of RNA-seq meta-analysis of psoriasis. Swindell et al. described psoriasis-specific as well as non-specific differentially expressed genes (DEGs). Interestingly, psoriasis-specific DEGs were expressed by keratinocytes and induced by IL-17A, whereas non-specific DEGs (in many other skin conditions) were expressed by inflammatory cells and induced by IFN-gamma and TNF [69].

## 5. MicroRNA

At the post-transcriptional level, miRNAs are recognized as being major players in gene expression regulation [101]. miRNAs are small, single-stranded non-coding RNAs which sequence is complement to specific 3ʹ untranslated regions (UTRs) of mRNA transcript and together with associated Argonaute proteins and other RISC components (RNA-induced silencing complex), silence the expression of target proteins and promotion of mRNA degradation [102,103]. 

Most circulating miRNAs are assembled in complexes with proteins that stabilize them [41]. Therefore, according also to our experience which is in line with the opinion of other authors, storage conditions and specimen processing procedures may have an impact on microRNA results [41]. However, some authors claim that in case of inflammatory diseases analysis of microRNA level in serum/plasma is not appropriate because high white blood cell counts may affect the results [104,105]. Moreover, expression profile of miRNAs is variable and depends on diseases entities [106]. In the case of RA many studies revealed correlation between different clinical parameters and dysregulation of expression analyzed miRNAs [41,107,108]. Many studies show that level of microRNA may help to predict therapeutic effects [70,109,110,111,112]. 

Some of microRNAs such as miR-146a, miR-199a, miR-155, miR-126, miR-21, miR-29, miR- 148/152, and miR-466l regulate the stability of mRNAs that encode TLR signaling molecules [113] which significance in autoimmune response has been abovementioned. Another important role in the autoimmune context is played by SMADs protein (TGFβ pathway and process of T cell differentiation) [114,115]. It is not surprising, that as transcription factors, SMADs may bind directly with the promoters of some microRNAs as miR-155, -216 [116], let-7a or let-7d [117] and upregulate or downregulate their expression as it has been noted in case of miR-24 [118] and miR-29 [119]. Moreover, it has been shown that SMADs associate with the Drosha microprocessor complex via p68 and facilitate the processing of pri-miR-21 into pre-miR-21 taking part in posttranscriptional processing of this miR-21 [120]. miRNAs have unique expression profiles in cells of the innate and adaptive immune systems and have pivotal roles in the regulation of both cell development and function [103]. Divergent miRNA expression profile has been determined in the synovial fibroblast of RA patients compared to this cell type in OA patients [121]. 

NGS techniques provide a possibility of global analysis of circulating microRNAs, discovery of novel miRNAs and reveal their polymorphism (isomiRs). Rare microRNA are more likely to be detected because this method is more sensitive [41]. 

Su et al. used miRSeq to determine the microRNA expression profiles of intracellular microRNA in peripheral mononuclear cells that were capable of differentiating between lupus patients with nephritis (LN) and those without nephritis. mir-125a-5p, miR-146a-5p, and mir-221-3p were found to be statistically significant in the screening study. Moreover, miR-146a-5p has been associated with the creatinine levels in lupus nephritis patients. Therefore, according to the authors, miR-146a-5p may serve as a useful specific biomarker for the detection of lupus nephritis among lupus patients in the future, regardless of serum albumin levels and spot urine protein/creatinine ratio. Additionally, it has been noted that LN patients after treatment were characterized by significantly elevated miR-125a-5p level [38]. Another study conducted by NGS technology also revealed over-expressed miR-146a in PBMC in SLE and primary Sjögren’s Syndrome [71]. miR-146 and miR-155 are among the first and most studied miRs for their multiple roles in the control of the innate and adaptive immune processes [122]. Hermann et al. confirm miR-146a anti-inflammatory function in the skin and indicate that in case of psoriasis, the expression of miR-146a/b is increased in response to disease-associated cytokines [123]. miR-146 is also a central element of an anti-inflammatory feedback loop in resident synovial fibroblasts. Its loss leads to increased joint destruction in a TNF-driven model of arthritis by specifically regulating the behavior of synovial fibroblasts. Transcriptome analysis of myeloid bone marrow cells and synovial fibroblasts by RNA sequencing allowed to find a high number of differently regulated genes in synovial fibroblasts between wide-type and miR-146a deficient cells [124]. Association of circulating miR-146a-5p in serum with prevalent knee OA in women has been described by Rousseau [125]. In RA patients, upregulated levels of miR-146a-5p, miR-155-5p and miR-132-3p in whole blood were observed in MTX responders. Therefore, microRNA levels might be useful marker of the treatment response [70]. Base on the high-throughput screening microRNA in CD14+ monocytes, another potential biomarker and treatment target for PsA has been proposed. Micro-RNA expression profile in PsA patients has been compared to the expression in psoriasis patients without arthritis (PsO), and healthy controls. Study indicated that miR-941 is associated with PsA activity and enhances osteoclastogenesis in PsA via WNT16 repression [47]. Chen’s et al. high-throughput research revealed that some miRNAs including miR-146a, miR-16 and miR-21 were over-expressed in pSS patients and as well in SLE group. Expression levels of miR-223-5p, miR-150-5p, miR-155-5p and miR-342-3p showed associations with the B cell proportions within peripheral blood mononuclear cells. Therefore, authors claim that the enhanced expression of this miRNA seems to be associated with systemic autoimmune processes, rather than a specific autoimmune disorder [71]. Another high-throughput microRNA study identified two microRNAs: miR-223-3p and miR-16-5p as markers of RA disease and may also shed more light on the pathophysiology of RA. These two microRNAs were significantly lower in the sera from early RA patients and healthy controls. The authors hypothesize that PAD activation during disease development decrease miR-16-5p level in blood and/or synovial fluid cells and increase cell proliferation [41]. Interestingly and worthy to mention that PADs proteins facilitate chromatin decondensation during NET formation (neutrophil cellular trap) which along with DNA and citrullinated histones are one of the significant factors during the developing autoimmunity [126]. Moreover, NGS combined with other sophisticated technology such as laser capture microdissection (LCM) provides a robust approach to exploration of variable compartments. One of the example is study conducted by Løvendorf et al. where authors revealed an association between the deregulated miRNAs in psoriatic plaque dermal inflammatory infiltrate and the miRNAs identified in the T cell subsets including the Th17 cells [44]. 

## 6. Microbiome

In 1909, Bailey stated that “chronic rheumatic arthritis” and “rheumatoid arthritis” (…) are inflammatory affections of a progressive character, probably caused by toxins elaborated by microorganisms [127]. Indeed for many years strategy of treatment with antibiotics seemed to be efficient [128,129,130] and in some cases is still recommended [131,132,133,134,135]. The concept of relationship between human microbiota with the health status has been repeated for many decades but for almost 100 years the only tool available to characterize bacteria was culture, which is highly inefficient. It has been estimated, that only 20% of intestinal bacteria can be cultured. Additionally, many of these bacteria require specialized media, and anaerobic culture which is also technically demanding [136]. Moreover, the term of microbiota proposed by Human Microbiome Project defined it as a communities not only bacteria, but also other microorganism including eukaryotes, archaea and viruses [137]. Therefore, culture-independent methods for characterizing the microbiota, together with a molecular approach provided a fundamental breakthrough in this field of research [138]. Depending on technical approach of microbiome study, different data are revealed. Metagenomics focus on sequencing of massive communities, define genetic content of all community members and reveal the potential function of the complete collection of microbes [139]. Metatranscriptomics required RNA isolation and define the actual physiological or metabolic status, without characterizing actual or direct enzymatic activity, of the community members. Enzymatic function expressed in a community is provided by metaproteomics approach whereas information of entire metabolites collection is provided by the metabolomic. Combining sequencing of whole genome and transcriptome analysis, so-called omics analysis might be conducted within whole community or single-cell [140]. 

**Figure 1:**
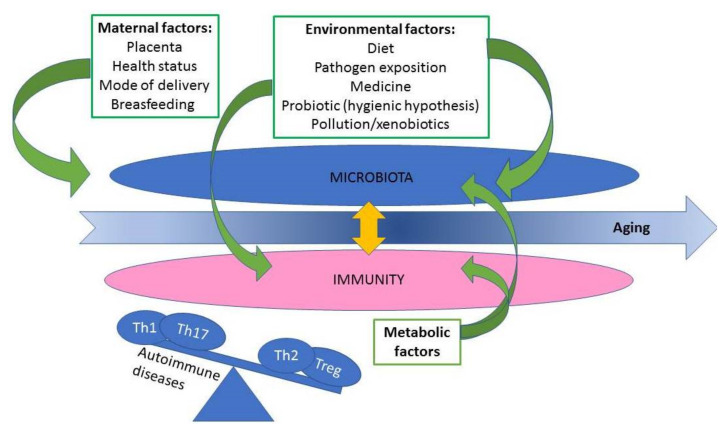
Scheme of the relationship between the microbiota, the immune system and other influencing factors. Changes in the microbiome may affect the balance of the lymphocyte subpopulation, contributing to the activation of the autoimmune cascade.

Skin, mucosa, and alimentary tract are a location of microbial ecosystem. However, every host and even hosts’ compartments are characterized by different communities of microorganisms, which is the result of diet, genotype, colonization history, stress and physiological state and physical and biochemical features of each location [141]. Changes to the microbiota can shift susceptibility to inflammatory, autoimmune, and infectious diseases [142]. Commensal microbiota shape immune cell development and phenotype [143], contributes to medication efficacy [144]. Gut microbes are necessary for developing normal host mucosal immunity. Animal model of germ free mice revealed severe mucosal, immune, and anatomic abnormalities [145,146]. Moreover, lower level *of IgA-producing plasma cells* [147], changed Th17:Treg balance in the lamina propria of the small intestine [148,149] and a skewed Th2 to Th1 ratio [150] (Figure 1). Evolution course adapted host to tolerate commensals by different mechanisms, such as Treg induction and IL-10 secretion [151] and increasing knowledge allows/enables to develop new probiotic formulation which helps to reduce symptoms of colitis, downregulate expression of proinflammatory mediators such as IL-6 and TNFα and upregulate IL-10 mRNA level and the number of Treg [152]. Additionally, intestinal microbiome takes part in nutrient processing [153] and has an impact on development of metabolic diseases. Conventialization of germ-free mice resulted in an increment in body fat content and insulin resistance within two weeks despite reducing food intake became obese and insulin resistance. It has been concluded that microbiota suppress Fiaf (Fasting-induced Adipose Factor), which is a circulating lipoprotein lipase inhibitor, and that suppression is essential for the deposition of triglycerides in adipocytes [154]. A number of metabolites from intestinal bacteria, such as short chain fatty acids (SCFA) facilitate Treg cell differentiation or their expansion in the gut [155]. The microbial metabolism of dietary tryptophan produces ligands of aryl hydrocarbon receptor (AhR) [115] which is of the most studied in the aspect of immunomodulation, reviewed in many articles [126,156,157,158,159]. Its role, including association with microbiome metabolism, has been proved not only in amelioration of inflammation in gastrointestinal tract [160,161,162] but also in the aspect of balanced skin physiology [163,164,165,166]. 

The idea of bacteria identification according to their ribosomal 16S DNA sequence has been introduced in 1977 [167] and until now in the era of completely different technology, this concept still constitutes a practical approach in microbiome research [144]. Nowadays, most researchers utilize 16S rRNA sequencing. Traditionally, the first step of 16S rRNA gene sequencing was to clone the gene, followed by sequencing of PCR amplicons by Sanger method. Currently, for the NGS method, the first step is DNA extraction, followed by 16S rRNA gene apposition and sequencing. Sequencing results are compared with the reference 16S rRNA sequences available in public databases. Avoiding the cloning stage is one of the major advantages of microbiome analysis by NGS method. Additionally, hundreds of samples can be simultaneously sequenced [168]. However, 16S rRNA sequencing has some limitations: primer choice is crucial and has an impact on the detection of certain microbial species and downstream analyses [142]. Designed primers use in the analysis are complement with the hypervariable regions (eg, V1–V3, V3–V4, V3–V5, V4). Due to the fact that in different studies selected primers are variable, it is one of the challenges in comparing the results between studies [144]. Moreover, the phylogenetic definition of analysed microbial species depends on available databases [142]. Additionally, the technique suffers from PCR biases and remains still relatively expensive and laborious [142]. Another challenge is the integration of statistics, bioinformatics, mathematical methods [155]. Very often subjects are not completely characterized by factors that are crucial for microbiome diversity, such as diet, medical comorbidities and treatment [144]. Sampling method of microbial material (swab, scrape, punch biopsy) and proper negative control or blank sample, e.g., consisted of environmental background are also necessary to define the accuracy of the data [169]. 

## 7. Conclusions

NGS approaches are necessary in the case of disorders like autoimmune diseases due to complex pathogenesis, difficult diagnosis and differentiated response to treatment. In this review, we tried to present the latest achievements in the field of transcriptome and microbiome research and genetic diagnosis of autoimmune diseases using NGS. 

Clinical application of NGS can provide genetic status not only for pathogenic variants of Mendelian and complex autoimmune diseases but also for pharmacogenomics variants and genetic risk factors such as HLA-alleles. Additionally, this technology provides a more comprehensive insight into different cell subpopulations of the immune system or body compartments. Together, this is extremely important for understanding the pathogenesis of autoimmune diseases. Identification for new molecular markers like microRNAs allows predicting the disease at the earliest stage and estimating therapeutic effects.

There are limitations in the NGS application but the technology improves leading to obtaining more accurate and faster data. In the near future, NGS is likely to become more popular for patients with suspected autoimmune diseases leading to earlier diagnosis and development of personalized effective treatments.

## Figures and Tables

**Table 1:** Studies on autoimmune connective diseases conducted by NGS in last 5 years.

CTD	Description/Notes	Reference
SLE	The repertoire of T-cells in clonal expansion of T cells may be efficiently measured by emerging of Β-loci of the T-cell receptor (TCR): peripheral blood (PB) in SLE patients revealed decreased and more uneven distributed repertoire of diversity in comparison to healthy controls (HC)	[36]
SLE	Significant upregulation (high diagnostic potential) of hsa_circ_0000479 was observed in PBMCs of SLE patients compared to HC in comprehensive expression analysis of circRNAs; effect of hsa_circ_0000479 affects SLE progression by modulation of the Wnt signaling and metabolic pathways	[37]
SLE	MicroRNA screening for early detection of lupus nephritis in SLE patients: statistically significant mir-125a-5p, miR-146a-5p and mir-221-3p. 146a-5p significantly correlated with markers of clinical biochemistry and proved to be one of the biomarkers for early detecting of lupus nephritis.	[38]
RA	Whole-genome SNP array and NGS analysis revealed strong association of HLA loci DRB1*03:01, DRB1*04:05, DQA1*03:03, and DQB1*04:01 in Taiwanese population with RA	[39]
RA	Changes in mitochondrial genome may be one of the factors leading to the RA progression; mitochondrial variants 12,308 A > G (gene tRNA^Leu(CUN)^) and 15,924 A > G (gene tRNA^Thr^) were found to be pathogenic.	[40]
RA	Serum level of miR-16-5p and miR-223-3p was significantly reduced in early RA patients compared to patients with established RA and HC; patients with established RA revealed upregulated miR-16-5p level in comparison to/with HC.	[41]
RA	Shared epitope (SE) alleles were studied as the most significant genetic predisposition locus in RA: SE allele-positive patients with RA showed reduced diversity of the T cell receptor (TCR) repertoire in memory CD4+ T cells compared to HC. RA activity and SE alleles negatively correlated with the diversity of TCR repertoire. Compensation of altered TCR repertoire diversity in RA may serve as a potential therapeutic target.	[42]
SSc	miRNA-26a-2-3 may be involved in pathogenic IFN signature in SSc monocytes	[43]
PsA	NGS was used to perform analysis of global microRNA expression in dermal and epidermal compartments: the epidermis and in the dermal inflammatory infiltrates of psoriatic skin deregulated compared with normal psoriatic skin and pool of deregulated miRNA was identified including miR-193b and miR-223 (described also as deregulated in PBMCs in patients with psoriasis).	[44]
PsA	The variant rs1061622 G (p.M196R) in TNFRSF1B was identified as strongly associated with the risk of psoriasis and the response to anti-TNF or anti-Il-12/Il-23 treatment. HLA-CW6-positive patients were more frequent carriers of rs1061622 G. Variant rs1061622 G was significantly more common in the non-responder group	[45]
Graves’ disease, RA, T1D	NGS-based typing of high-resolution HLA gene polymorphisms in Japanese population revealed significant nonadditive effects of HLA-DPB1*05:01 and HLA-DPB1*02:02 alleles on the risk of Graves’ disease; HLA-DQβ1 at rs9273367 in LD with HLA-DQβ1 Ile185 (r2 = 0.81) with type 1 diabetes, RA significantly associated with HLA-DRB1, HLA-DQA1	[46]
PsA	MiRNA expression pattern in CD14 + monocytes was investigated in PsA patients. MiR-941 was shown to enhance osteoclastogenesis in PsA through repression of WNT16. The miR-941 is considered as a possible biomarker and target for the PsA treatment since its expression level in CD14+ monocytes is correlated with disorder activity.	[47]
RA, SjS	A repertoire of expressed BCRs was analyzed in RA and Sjögren’s syndrome cohorts, focusing on the main antigen-binding IgG variable heavy (IgGHV) region. Both cohorts expressed significantly more IgG+ve BCR sequences with fewer than five mutations referred as hypomutated (or IgGhypoM). The prevalence of IgGhypoM expressing B cells may play a crucial role in driving chronic inflammation in systemic autoimmunity.	[48]
BD	NOD2 seems to be the main contributor in the pathogenesis of Behcet’s disease.	[49]

SLE—Systemic Lupus Erythematosus; RA—Rheumatoid Arthritis; SSc—Systemic Sclerosis’ PsA—Psoriasis; T1D—Type 1 diabetes; SjS—Sjögren syndrome; BD—Behçet’s disease. An asterisk [*] in HLA allele name indicates that the allele was typed using molecular methods.

**Table 2:** Summary of the clinical NGS applications described in the review.

	NGS Applications in Clinical Use	References
	**Panels, whole-genome and -exome sequencing in diagnostic**	
1.	Example of genes evaluated as important in AID by Rusmini	[50]
2.	PTEN mutation and example of genes evaluated as important in vasculitis, inflammation by Mauro	[51]
3.	ACP5 mutation associated with SLE, Sjogren Syndrome, inflammatory myositits	[52]
4.	AIRE in Autoimmune Polyendocrine syndrome Type I	[53]
5.	NLRP3 in Cryopyrin-associated periodic syndrome (CAPS)	[54]
6.	NOD2 mosaicism in Blau Syndrome	[55]
7.	homozygous mutation in TREX1 (R97H) in SLE	[56]
8	BTNL2 variant associated with RA	[57]
9.	CLC (rs146776010),FBXL14 (rs117331652), DCLRE1C (rs772438042) or NOTCH1 (rs758642073) may have direct impact on immunology system and possible development of autoimmune disorders.	[58]
10.	Copy number loses in HIST2H2AA3), HIST2H2AA4, HIST2H3A, HIST2H3C, HIST2H4A and HIST2H4B in SLE	[59]
11.	mutations in C1S, DNASE1L3, DNASE1, IFIH1, and RNASEH2A, C1QC in SLE	[60]
12	DNASE1L3 and HDAC7 in SLE	[61]
13	NRAS and PI3CKD gene with TNFAIP3 should be considered as candidates gene for testing in children with SLE with lymphoproliferation, particularly for male patients with renal and hematologic involvement and recurrent fevers	[62]
	**Human leukocyte antigens as a diagnostic tool**	
1.	DQB1*04:02:01 and DR8-haplotype frequencies significantly higher in patients with oligoarthritis but not systemic juvenile idiopathic arthritis; protective effect against systemic and oliarticular JIA of DQA1*05:01:01 and DQB1*02:01:01	[63]
2.	Asp at HLA-B position 9 and Phe at HLA-DPB1 position 9- predisposition to RA development; Val and Leu at HLA-DRB1 position 11 association with a risk of a higher rate of radiographic progression and predisposition to RA development	[64]
3.	HLA-B27 association with ankylosis spondylitis	[65]
4.	HLA-DQß1 in position 57, 13 and 71—higher risk of Type 1 Diabetis	[66]
	**RNA-seq**	
1.	RNA-seq for the prediction of medical treatment	[67,68,69]
	**micro-RNA**	
1.	miR-223-3p and miR-16-5p biomarkers of RA	[41]
2.	miR-146a-5p associated with carnitine level in lupus nephritis and may be a potential diagnostic biomarker of lupus nephritis among lupus patients	[38]
3.	Higher levels of miR-146a-5p, miR-155-5p and miR-132-3p might be useful marker of the methotrexate treatment response.	[70]
4.	miR-941 is associated with PsA activity	[47]
5.	Overexpression of miR-146a, miR-16 and miR-21 in primary Sjogren Syndrome and SLE patients	[71]

NLRP3—family pyrin domain containing 3; BTNL2—butyrophilin-like 2 gene; CLC—Charcot-Leyden crystal protein, FBXL14—F-box and leucine-rich repeat protein 14, DCLRE1C—DNA cross-link repair protein 1C, NOTCH1—NOTCH receptor 1; HIST2H2—histone cluster 2 H2A/H3C/H4A/H4B family member A3/A4/3C/4A/4B; C1QC—Complement C1q C Chain; HDAC7—histone decetylase 7. An asterisk [*] in HLA allele name indicates that the allele was typed using molecular methods.

## Data Availability

Not applicable.
